# The Organon and Materia Medica Club of the Bay Cities of California

**Published:** 1895-04

**Authors:** W. E. Ledyard

**Affiliations:** Secretary


### *THE ORGANON* AND MATERIA MEDICA CLUB OF THE BAY CITIES OF CALIFORNIA.

### October 18th, 1894.

Sections 39 to 45 of *The Organon* were read.

Dr. J. M. Selfridge reported a case of "chills and fever" which had been treated unsuccessfully by Quinine. Natrum-mur.$^{200}$ was given in the apyrexia. The succeeding paroxysm was lighter. When the attack again became severe he repeated the dose after the paroxysm, and in two weeks the patient had had no return of the chills, and merely complained of bone pains.

Dr. Lilienthal had a case of chills suppressed by Quinine. Nausea called for Ipecac, which brought back the original chills, which were then cured by Eupatorium-perfoliatum.

The Secretary reported a case of asthma, caused by the suppression of "chills and fever" by Quinine. The first chill occurred in 1846, recurring every other day in the afternoon. After coming to California had the attacks every spring and fall (Lach. and Sepia). In 1867 had mountain-fever; slight chill, with stretching and yawning (Ars., Bry., Caps., Cim.,Elat., Eup-perf., Laur., Marum, Mur-ac., Polyp.); then high fever with great thirst, followed by sweat. At first he took twenty-five grains of Calomel; then Quinine, until he had ringing in the ears. He used to buy Quinine by the ounce. After taking the Quinine the chills disappeared, but asthma took its place, the paroxysms gradually increasing in severity. Taking into consideration the overdosing with Quinine; the fact that the original chill recurred every other day, in the afternoon; that the fever was at that time high and attended with great thirst; and that these symptoms, together with difficult expectoration at two A. M., all pointed to Quinine, on December 20th, 1893, he received one dose of Chin-sulph.$^{200}$ dry. This was repeated on January 3d, 1894. On January 8th the patient reported "creeping chills" on the night of the 3d (having taken the second dose of Chin-sulp. that day). These chills kept coming and going frequently night and day, and were followed by fever; sweat, when coughing or on exertion; complains of the same yawning and stretching as when he had chills and fever. On 11th, 12th, and 13th, high fever, from five P. M. until bed-time. Since that time the asthma has been comparatively light, and his general health has been much better.

Sulphur is the indicated remedy for the asthma in this case, and he had received a dose at long intervals, ranging from the 200th to the 500 M potency.

NOVEMBER 2d, 1894.

*The Organon* was read and discussed from §§ 46 to 52, inclusive.

Dr. McNeil—These cases show that Nature cures a disease by another always similar.

Dr. J. M. Selfridge put the question—" Does not Hahnemann recognize cow-pox as a preventive of small-pox ?"

Dr. McNeil replied that Hahnemann says that cow-pox is an important prophylactic of small-pox on account of its homœopathicity. Hebra states that the vesicle of the cow-pox is an exact simillimum of the small-pox. He (Dr. McNeil) thinks nothing more clearly established than the efficiency of cow-pox in modifying or preventing small-pox. Small-pox, before cow-pox was used, made terrible ravages. He alluded to the disastrous effects resulting from it in Mexico. He is of the opinion that Homœopathy should claim the benefits of vaccination as its own.

Dr. J. M. Selfridge remarked that cow-pox and small-pox were considered identical by some.

Dr. McNeil—It has been stated that after inoculating the cow with small-pox a malignant form of the disease was produced.

Dr. J. E. Lilienthal—People are now beginning to talk of again vaccinating with humanized virus. They have found that the cow-pox virus is not reliable.

Dr. C. M. Selfridge mentioned two cases in which sloughing occurred after vaccination.

Dr. Augur would like to know about the effect of using Variolinum and Vaccininum in the treatment of small-pox.

Dr. Lilienthal stated that Dr. Raue had used both successfully.

Dr. McNeil said that the late Dr. Pease reported a case to the Health Officer. He (Dr. Pease) gave Vaccininum, and reported the case cured in a few days. The Health Officer thereupon concluded that he (Dr. Pease) must have been mistaken in his diagnosis.

Dr. Wilson stated that Dr. Breyfogle gave Vaccininum successfully.

Dr. McNeil claimed the right to ask how long will Vaccininum, Variolinum, or Melandrinum protect?

Dr. Lilienthal retorted with the question—"How long will vaccination protect?"

Dr. McNeil asserted that after vaccination for eight years there was not a single death from small-pox in the Prussian army.

Dr. Lilienthal remarked that it was also on record that many soldiers who had been vaccinated afterward contracted small-pox.

Dr. J. M. Selfridge stated that when he had vaccinated with virus, tinged with blood, he had seen bad effects.

Dr. McNeil remarked that the Martins vaccinated new heifers every year.

Dr. J. M. Selfridge stated that the virus could be preserved indefinitely by cold.

Dr. McNeil vaccinated successfully two weeks ago with virus two or three months old. The Old School in their anti-toxine, etc., are homœopathic. They claim that they can cure ninety-five per cent. of cases of diphtheria.

Dr. Lilienthal asked the question, "If we accept it as isopathic, must we not believe in the bacterial theory?"

Dr. J. M. Selfridge does not believe that the baccilla is the cause of the disease. Anything that prevents the elimination from the system of the ptomaines, which are the natural products of decay, will cause disease and give rise to stupor, fever, etc. Bacteriologists claim that the ptomaines are the product of the baccillus, and that these spread through the system. Poisons that should be eliminated are retained in the system. He admits that if the baccillus is injected under the skin it will spread and germinate.—Klebs Loefler.

Dr. Lilienthal—In a healthy throat the baccillus is frequently found, and still there is no diphtheria. The system has to be in a condition to receive the baccillus.

Dr. McNeil—Dr. W. P. Wesselhœft said that an early diagnosis in consumption can be made by finding the baccillus.

Dr. J. M. Selfridge—You will not find the baccillus in consumption until decomposition has set in. He would make a physical examination, and would like to corroborate it by the examination of the sputum.

Dr. Augur reported a case of diphtheria in a child nine months old. The child had inoculated its face so that there were several pustules or rodent ulcers of the size of a pea. One side of the face was much swollen. In the throat there were large diphtheritic patches. There was a tendency to throw the head back, inclining to opisthotonos. Liquids regurgitated through the nose. Belladonna cured the case, although the child had been treated by an Old School physician for four days, and had been pronounced incurable.

Dr. Lilienthal prescribed carefully for a sore throat, which afterward proved to be syphilitic. No improvement following he gave Iodide of Potassium, a saturated solution, three times a day, and cured him.

The Secretary suggested that possibly the above was a suppression and not a cure.

Dr. J. M. Selfridge—The effects of the suppression might not show themselves for years.

Dr. Lilienthal—It is homœopathic if it cures, whether in the saturated solution or in the millionth potency.

Dr. McNeil—The high potency will cure every time if you give the right remedy.

(Signed) W. E. LEDYARD,
*Secretary.*

